# Opposing effects of oxidative challenge and carotenoids on antioxidant status and condition-dependent sexual signalling

**DOI:** 10.1038/srep23546

**Published:** 2016-03-22

**Authors:** Oldřich Tomášek, Barbora Gabrielová, Petr Kačer, Petr Maršík, Jana Svobodová, Kamila Syslová, Michal Vinkler, Tomáš Albrecht

**Affiliations:** 1 Institute of Vertebrate Biology, v.v.i., The Czech Academy of Sciences, Květná 8, Brno 603 65, Czech Republic; 2 Charles University in Prague, Faculty of Science, Department of Zoology, Viničná 7, 128 44 Praha 2, Czech Republic; 3 Institute of Chemical Technology Prague, Technická 5, 166 28 Praha 6, Czech Republic; 4 Institute of Experimental Botany, v.v.i., The Czech Academy of Sciences, Rozvojová 313, 165 02 Praha 6, Czech Republic; 5 Department of Quality of Agricultural Products, Faculty of Agrobiology, Food and Natural Resources, Czech University of Life Sciences, Kamýcká 1176, 165 21 Praha 6, Czech Republic; 6 Department of Ecology, Faculty of Environmental Sciences, Czech University of Life Sciences, Kamýcká 1176, 165 21 Praha 6, Czech Republic

## Abstract

Several recent hypotheses consider oxidative stress to be a primary constraint ensuring honesty of condition-dependent carotenoid-based signalling. The key testable difference between these hypotheses is the assumed importance of carotenoids for redox homeostasis, with carotenoids being either antioxidant, pro-oxidant or unimportant. We tested the role of carotenoids in redox balance and sexual signalling by exposing adult male zebra finches (*Taeniopygia guttata*) to oxidative challenge (diquat dibromide) and manipulating carotenoid intake. As the current controversy over the importance of carotenoids as antioxidants could stem from the hydrophilic basis of commonly-used antioxidant assays, we used the novel measure of *in vivo* lipophilic antioxidant capacity. Oxidative challenge reduced beak pigmentation but elicited an increase in antioxidant capacity suggesting resource reallocation from signalling to redox homeostasis. Carotenoids counteracted the effect of oxidative challenge on lipophilic (but not hydrophilic) antioxidant capacity, thereby supporting carotenoid antioxidant function *in vivo*. This is inconsistent with hypotheses proposing that signalling honesty is maintained through either ROS-induced carotenoid degradation or the pro-oxidant effect of high levels of carotenoid-cleavage products acting as a physiological handicap. Our data further suggest that assessment of lipophilic antioxidant capacity is necessary to fully understand the role of redox processes in ecology and evolution.

Over the last four decades there has been a growing interest in sexually selected traits as honest indicators of individual quality and health^1^,^2^; however, mechanisms linking ornament expression to individual condition remain elusive^3^,^4^. Increasingly, redox processes (and oxidative stress in particular) have been hypothesised as providing this link and thereby maintaining honesty in many sexual signals^4^,^5^,^6^. Reactive oxygen and nitrogen species (ROS), the source of oxidative stress and damage, are inevitable and ever-present by-products of an oxidative metabolism. ROS can also arise through immune activation^7^ or environmental pollution^8^. The need to prevent oxidative stress and maintain redox homeostasis, therefore, could provide a universal mechanism linking ornament expression to immune activation and other metabolically demanding processes (e.g. stress response, moulting or sperm production)^5^,^6^.

Carotenoids are traditionally considered to be important antioxidants due to their ability to quench ROS *in vitro*^9^. A trade-off in allocation of carotenoids between ornamentation and antioxidant defence has therefore been proposed as a possible mechanism maintaining signalling honesty (the ‘allocation trade-off hypothesis’)^5^,^10^,^11^. The importance of carotenoids as antioxidants *in vivo* has recently been questioned^12^,^13^,^14^, however, resulting in the emergence of several alternative hypotheses. The ‘protection hypothesis’ assumes no carotenoid antioxidant function, proposing instead that redox homeostasis is signalled through ROS-induced carotenoid oxidation and cleavage, which results in colour loss that can be prevented by an efficient antioxidant system^12^. An extension of this hypothesis is the ‘handicap hypothesis’, which proposes that high levels of carotenoids may directly aggravate oxidative stress through pro-oxidant activity of carotenoid cleavage products, thereby posing a direct physiological handicap^15^,^16^. More recently, the structural and functional similarities between carotenoids and ubiquinones have prompted the hypothesis that synthesis of red carotenoids could occur within the inner mitochondrial membrane (IMM) of hepatocytes, with carotenoids, redox-coupled with ubiquinones, displaying antioxidant and modulatory functions in the electron transport chain. The hypothesis proposes that the synthesis of red carotenoids could be linked to the maintenance of the IMM potential, thereby linking ornament expression not only to redox homeostasis but also to efficacy of mitochondrial respiration (the ‘IMM carotenoid oxidation hypothesis’)^17^.

Clearly, knowledge of the actual role of carotenoids in redox homeostasis is crucial if we are to test hypotheses on the mechanisms ensuring carotenoid-based signalling honesty^18^. Studies into carotenoid antioxidant function *in vivo* have provided mixed results, however, leading to an overall low association of carotenoid levels with antioxidant capacity in a recent meta-analysis^13^. We suggest that the current controversy over carotenoid antioxidant function could stem from the use of inappropriate methods for antioxidant capacity assessment. Due to their lipophilic nature, carotenoids are located in the interior of an organism’s lipid bilayers and other lipid compartments. As lipid bilayers (e.g. mitochondrial membranes) are considered a major source of highly damaging ROS^9^, carotenoids have great potential to play an important role in redox homeostasis. Most commonly-used antioxidant assays (e.g. FRAP, ORAC, OXY-Adsorbent test, TEAC or TRAP), however, measure antioxidant capacity in aqueous media only, which renders them unsuitable for evaluation of lipophilic antioxidants such as carotenoids^19^,^20^. With this in mind, we decided to use a novel marker of lipophilic antioxidant capacity, i.e. the ratio of *ZE*- and *EE*-stereoisomeric forms of hydroxyoctadecadienoic acid (HODE). *ZE*- and *EE*-HODE are formed as oxidation products of linoleic acid, with the proportion of *ZE*-HODE to total HODE (*ZE*/tHODE) increasing with higher concentrations of hydrogen-donating lipophilic antioxidants such as vitamin E or coenzyme Q, thereby providing an integrated measure of their activity *in vivo*^21^,^22^. As carotenoid ROS-scavenging activity is most likely mediated through radical addition rather than hydrogen donation^9^,^23^, carotenoid antioxidant action is probably not measured directly by *ZE*/tHODE. Despite this, the lipophilic nature of this antioxidant-capacity measure makes it far better candidate for detecting a possible effects of carotenoids on an organism’s antioxidant system than traditional hydrophilic-based assays.

In this study, our aim was to assess redox-based hypotheses of carotenoid-based signalling honesty by testing the importance of carotenoids for redox homeostasis and the role of redox homeostasis in constraining carotenoid-based signal expression. Using a 2 × 2 factorial design, we analysed the effects of oxidative challenge exerted by diquat dibromide and carotenoid supplementation on beak colouration, circulating carotenoid levels and redox homeostasis in adult zebra finch (*Taeniopygia guttata*) males. Diquat dibromide, a bipyridyl compound known to generate superoxide anions *in vivo* through redox-cycling, has recently been recognised as a convenient oxidative stress inducer in ecological studies^24^,^25^, as well as in laboratory models of Parkinson’s disease^26^,^27^,^28^. If redox homeostasis really constrains signal expression, beak colouration should be reduced following diquat treatment. Since a change in oxidative stress intensity can be manifested as either a change in oxidative damage or in antioxidant activity (or, indeed, both together)^29^, we predict that carotenoid supplementation will result in (a) a reduction in oxidative damage and/or activity of antioxidants other than carotenoids due to their reduced need^18^,^29^,^30^,^31^ (lowered *ZE*/tHODE ratio) if carotenoids act as antioxidants, (b) an increase in either one or both of these parameters if carotenoids act as pro-oxidants, or (c) no effect on either parameter if carotenoids have no influence on redox homeostasis.

## Results

### Effect of oxidative challenge and carotenoid intake on ornament expression, plasma carotenoids and body mass

**Figure 1:**
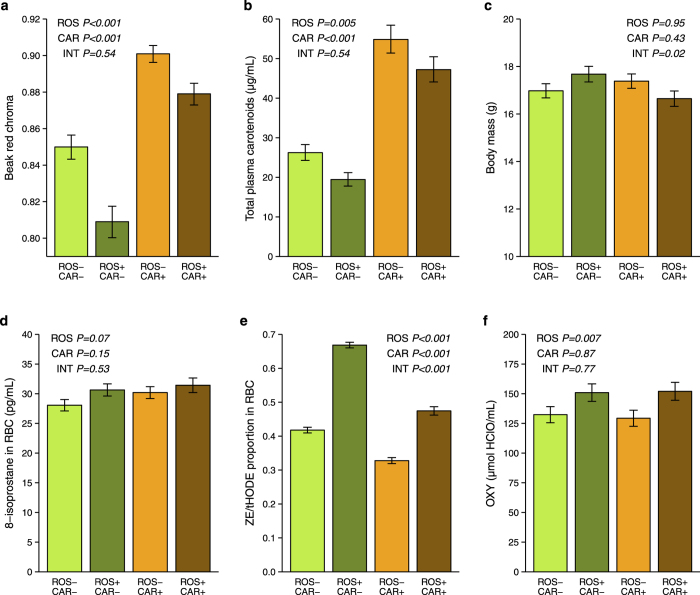
Effects of oxidative load and carotenoid intake on beak colouration, circulating carotenoids, body mass and blood redox state. Birds were exposed to either low (ROS−) or high oxidative load (ROS+) and to either low (CAR−) or high carotenoid intake (CAR+). Bars represent group means controlled for pre-experimental values and their standard errors obtained from the models in Table 1. Shown are model *P*-values for the main effects of oxidative challenge (ROS) and carotenoid intake (CAR), as well as their interaction (INT). (**a**,**b**) Both, beak red chroma (higher values = more saturated red colour) and total plasma carotenoids were reduced by oxidative challenge and enhanced by high carotenoid intake. (**c**) Neither treatment factor had a significant effect on body mass, though their interaction resulted in body mass in ROS + CAR + group that was lower than would be expected if both effects were additive. None of the treatment groups, however, differed from the control (Tukey’s post-hoc test on change scores: *P* ≥ 0.379). (**d**–**f**) Oxidative challenge elicited a marked increase in both, lipophilic (*ZE*/tHODE) and hydrophilic (OXY) antioxidant capacity, resulting in marginally insignificant increase in blood oxidative damage (8-isoprostane). Carotenoids counteracted the effect of oxidative challenge on the activity of other lipophilic antioxidants (shown as reduced *ZE*/tHODE ratio) while having no effect on oxidative damage levels. On the other hand, hydrophilic antioxidant capacity was unaffected by lipophilic carotenoids.

Diquat-induced oxidative challenge of experimental males resulted in a significant decrease in beak red chroma supporting the role of redox homeostasis in maintenance of signalling honesty (Table 1; Fig. 1a). On the contrary, signal intensity was significantly enhanced following high carotenoid intake. These effects on beak pigmentation were additive with no significant interaction. Analysis of beak hue and UV chroma produced qualitatively similar results showing decreased redness and increased UV reflectance following oxidative challenge and the opposing effect of high carotenoid intake (see Supplementary Table S3).

Similar additive effects were observed in the case of total plasma carotenoids, with free oxidative challenge decreasing and high carotenoid intake increasing circulating carotenoid levels (Fig. 1b).

The treatment factors interacted in their effects on body mass, which increased slightly following either high oxidative load or high carotenoid intake, though this effect was inhibited when both treatments were combined. Change in body mass was not significant in any of the treatment groups, however, when compared to the control group (Tukey’s post-hoc test on change scores: *P* ≥ 0.379).

### Effect of ROS exposure and carotenoid intake on blood redox state

Interestingly, neither oxidative challenge nor carotenoid intake affected total oxidative damage measured as 8-isoprostane in red blood cells (RBC), though there was a marginally insignificant increase in 8-isoprostane following oxidative challenge. There was, however, a significant increase in RBC hydrogen-donation mediated lipophilic antioxidant capacity (*ZE*/tHODE) in response to oxidative challenge (Fig. 1c). In contrast, increased carotenoid intake provoked the opposite effect, resulting in a significant reduction in *ZE*/tHODE. There was also a significant interaction between the treatments, with substantial inhibition of the diquat-induced increase in *ZE*/tHODE by high carotenoid intake suggesting an *in vivo* antioxidant effect for carotenoids. Hydrophilic plasma antioxidant capacity, measured using OXY, was also elevated in response to oxidative challenge (Fig. 1d) though, in contrast to lipophilic *ZE*/tHODE, it was not affected by differing carotenoid intake.

## Discussion

In our study, oxidative challenge and carotenoid intake had opposing effects on beak ornamentation, plasma carotenoid levels and lipophilic antioxidant capacity in RBC. Despite the considerable adverse effect of oxidative challenge on both signal intensity and circulating carotenoid levels, it only resulted in a marginally insignificant increase in blood oxidative damage. Although blood redox state is usually used as an estimate of the body’s overall redox state, we cannot completely rule out elevated oxidative damage in particular organs such as the liver or kidneys^32^. Nevertheless, oxidative challenge elicited a marked increase in both hydrophilic and lipophilic antioxidant capacity, suggesting mobilisation of antioxidants to prevent increasing oxidative damage^24^,^29^. Since increased antioxidant capacity accompanied with stable (or increased) oxidative damage should be interpreted as higher oxidative stress^29^, this result support the pro-oxidant effect of diquat treatment. Importantly, carotenoids counteracted the effect of oxidative challenge on hydrogen-donating lipophilic antioxidants, as demonstrated by substantial inhibition of a diquat-induced increase in *ZE*/tHODE ratio by high carotenoid intake, while having no effect on oxidative damage levels. As radical addition has been proposed as the main carotenoid antioxidant mechanism^9^,^23^, rather than hydrogen donation, which is measured by *ZE*/tHODE^21^, the inhibition of the diquat-induced increase in ZE/tHODE by high carotenoid intake probably reflects down-regulation of hydrogen donating lipophilic antioxidants^29^ caused by the superoxide arising from diquat redox cycling^33^ being effectively quenched by the carotenoids^34^. The tendency of an organism to down-regulate other antioxidant mechanisms, rather than to reduce oxidative damage levels, has previously been documented following supplementation of antioxidants such as vitamins C or E^30^,^31^. As reduced activity of other antioxidants, together with maintenance of stable oxidative damage is suggestive of reduced oxidative stress^29^ our data support the antioxidant function of carotenoids *in vivo*.

Despite its substantial effect on lipophilic-based ZE/tHODE, carotenoid intake did not affect the hydrophilic-based OXY assay. The differing responses of these two antioxidant measures clearly demonstrate that the effect of carotenoids on an antioxidant system is missed when hydrophilic-based assays are used. As antioxidant status is usually assessed with hydrophilic-based methods, such as FRAP, ORAC, OXY, TEAC or TRAP^19^,^20^, this provides a possible explanation for the overall weak association of carotenoid levels with antioxidant capacity that was reported in a recent meta-analysis^13^.

The antioxidant effect of carotenoids observed in our study is inconsistent with the ‘handicap hypothesis’^16^, which assumes a pro-oxidant effect at high carotenoid concentrations, especially in the context of elevated oxidative stress. In effect, this hypothesis predicts that high carotenoid intake elicits either a significant increase in oxidative damage or a protective increase in *ZE*/tHODE in order to prevent such oxidative damage. In this study, however, we observed no adverse effect of carotenoids on blood redox state under low or high oxidative load, despite using the maximum absorbable carotenoid dose for zebra finches^35^. While (to the best of our knowledge) carotenoid intake in wild zebra finches remains unknown, work on house finches (*Haemorhous mexicanus*) suggests that wild diet in granivorous songbirds can contain as much as 90–100 μg/g of carotenoids^36^. As the 200 μg/g carotenoid dietary dose used in our study only resulted in a small increase in plasma carotenoid levels over the 100 μg/g dose in a previous study due to a plateau in carotenoid absorption at high concentrations^35^, we suggest that our dose resulted in body carotenoid concentrations that were at the upper limit of that which songbirds may experience in the wild. Even if such a dose resulted in body concentrations of carotenoids that were higher than that experienced in the wild, the lack of any pro-oxidant action suggests that honesty of carotenoid-based sexual signals is not maintained through a physiological handicap mechanism based on the pro-oxidant effect of high carotenoid levels, as proposed by the ‘handicap hypothesis’^16^. The observed antioxidant effect of carotenoids is also inconsistent with the ‘protection hypothesis’, which assumes no carotenoid effect on redox state^12^.

Our results are in accordance with the ‘allocation trade-off hypothesis’^5^,^10^,^11^, as a key assumption of this hypothesis is the antioxidant function of carotenoids. Allocation trade-off is further supported by the fact that, following oxidative challenge, a decrease in signal intensity was accompanied by an increase in antioxidant capacity but only marginally increased oxidative damage. Rather than simply being an effect of oxidative stress, therefore, reduced ornament expression appears to result from an increased investment in self-maintenance via reallocation of resources to antioxidant defence^24^,^29^. Such reallocation of resources may apply not only to antioxidants (possibly including carotenoids), but also other micronutrients necessary for carotenoid metabolism (e.g. cofactors) or energy, as carotenoid absorption and conversion are believed to be energy demanding^37^,^38^.

Our findings supporting carotenoid antioxidant function are also consistent with the recently proposed ‘IMM carotenoid oxidation hypothesis’^17^. Under this hypothesis, reduced signal expression following diquat exposure is interpreted as the result of reduced red keto-carotenoid production due to mitochondrial dysfunction, rather than resource reallocation to antioxidant defence. Red keto-carotenoid synthesis, however, is believed to take place in the beak tissue in zebra finches^39^ and not in the liver mitochondria, as assumed by the ‘IMM carotenoid oxidation hypothesis’^17^. In addition, red keto-carotenoid synthesis has recently been proposed as being catalysed by cytochrome P450 2J2 in zebra finches^40^. Inferring from human studies, this cytochrome is probably located in the endoplasmic reticulum and not in mitochondria^41^. These two pieces of evidence suggest that the ‘IMM carotenoid oxidation hypothesis’ may be a less likely explanation for carotenoid-based signalling honesty, at least in this model species.

The reduced ornament expression following diquat exposure observed in our experiment is the first demonstration of an adverse effect of a redox-cycling agent on a carotenoid-based signal in adult animals. As induction of oxidative stress is a primary mechanism of diquat toxicity^28^,^33^,^42^,^43^, this result supports the role of redox homeostasis as a major constraint ensuring honesty in carotenoid-based sexual signalling. No loss in body mass was observed in our diquat-exposed birds, indicating that the reduced ornamentation was not a consequence of a reduced food intake or impaired total intestinal absorption due to a possible diquat toxic effect on the intestinal wall. Our results therefore support the idea that energetically demanding and stressful conditions^44^, immune activation^35^,^45^ or environmental pollution (e.g. urban environment, heavy metals or pesticides)^46^,^47^ could reduce carotenoid-based ornamentation through elevated oxidative stress^5^.

Only four previous studies have directly tested the effect of oxidative status on expression of carotenoid-based ornament by either specifically increasing oxidative load through redox-cycling agents^25^,^48^,^49^ or reducing antioxidant protection^50^, and these produced contrasting results. In these studies, while paler beak and skin pigmentation developed following redox state disruption in juvenile red-legged partridges (*Alectoris rufa*)^25^, no effect was observed on feather colouration was observed in moulting adult great tits (*Parus major*)^48^, greenfinches (*Carduelis chloris*)^50^ or house finches (*Haemorhous mexicanus*)^49^. One possible explanation for this discrepancy could be a difference in trade-off solutions between developing and adult birds^25^, with the latter investing more in reproduction than in self-maintenance. Our demonstration of such an effect in adult sexually active zebra finch males, however, does not support this explanation. To date, the adverse effect of oxidative challenge has only been observed on bare parts only^25^, perhaps suggesting a higher sensitivity to oxidative stress in the more dynamic bare parts, which can change colour over a period of just a few weeks^45^,^51^, compared to semi-static feather ornamentation. Hence, future studies should test for possible differences in honesty mechanisms and signalling content between semi-static and dynamic carotenoid-based signals and explore possible differences in these mechanisms between different taxa and life-histories.

## Conclusion

Using a novel measure of lipophilic antioxidant capacity, we demonstrate that carotenoids are able greatly inhibit the effect of oxidative challenge on redox state, thereby supporting the recently questioned antioxidant function of carotenoids *in vivo*. This result is inconsistent with both the ‘protection’ and the ‘handicap hypotheses’, but provides circumstantial support for the ‘allocation trade-off hypothesis’, as carotenoid antioxidant function is a key assumption of the latter. Other mechanisms, such as regulation of carotenoid absorption and/or metabolism cannot be ruled out, however, and studies using isotopic labelling of ingested carotenoids will probably be needed to ascertain the relative involvement of allocation trade-off and other mechanisms in carotenoid-based signalling honesty.

We further demonstrate that the antioxidant effect of carotenoids is missed when a common hydrophilic-based antioxidant assay is used. Our data also demonstrate an organism’s impressive ability to adjust its antioxidant mechanisms and maintain stable levels of blood oxidative damage, despite large fluctuations in oxidative load or carotenoid intake. As a combination of oxidative damage markers and hydrophilic-based antioxidant assays is usually used for redox state assessment, these two observations provide a possible explanation for the generally weak support for carotenoid antioxidant function *in vivo* reported in a recent meta-analysis^13^. We suggest that the assessment of lipophilic antioxidant capacity will allow for a deeper understanding of redox processes in lipophilic compartments, such as lipid bilayers, and their ecological and evolutionary importance.

## Material and Methods

### Ethical approval

All experimental procedures were conducted in accordance with the Guidelines for Animal Care and Treatment of the European Union, and were approved by the animal care and ethics representatives of the Faculty of Science, Charles University in Prague, and The Czech Academy of Sciences (No. 041/2011).

### Model species

The zebra finch is a traditional model species, widely used in studies exploring links between individual condition and expression of sexual signals^18^,^35^,^51^,^52^. Both sexes have carotenoid-based beak pigmentation, which is sexually dimorphic and is known to play an important role in mate choice^53^. Redness of the beak has been reported as predicting future survival, reproductive success^54^ and immune reactivity^55^. Condition dependence in beak colouration has been demonstrated experimentally through a reduction in colouration following exercise, reduced food intake^56^, immune activation^35^ or cold stress^44^.

### Subjects and housing

Adult zebra finch males (*n* = 60) were housed individually in cages (0.6 × 0.4 × 0.4 m), at an indoor facility at the Institute for Vertebrate Biology of The Czech Academy of Sciences (Studenec, Czech Republic) from November 2010 on. All males were one to one-and-a-half years old at the time of the experiment. The birds were provided with millet seed, cuttlefish bone, grit with crushed shell and water *ad libitum*. Four weeks before the start of the experiment, millet seed was replaced by hulled millet seed, which was used for carotenoid supplementation during the experiment (see below). Photoperiod was set at 10:14 (light:dark) during winter, and gradually changed to 14:10 over April and May 2011. Eight females in separate cages were added to the experimental room at the end of May in order to stimulate breeding condition in males through visual and vocal contact. No other changes in social structure were made thereafter.

### Experimental design

We manipulated the level of oxidative load (ROS) and carotenoid intake (CAR) in a fully factorial design (2 × 2), with two levels (low and high) for each factor. Adult males were randomly assigned to four treatment groups (ROS−CAR−, ROS + CAR−, ROS−CAR + or ROS + CAR + ) with fifteen birds in each group. The experimental treatments lasted 10 weeks, from July to August 2011.

Diquat dibromide (Reglone 200 g/L, Syngenta, UK), a chemical compound known to generate oxidative stress *in vivo* through production of superoxide anions^24^,^25^,^26^,^27^, was used for the controlled increase in free radical exposure (ROS + groups). A fresh diquat solution was prepared each day and added to the drinking water. Based on a preliminary experiment (four groups of six birds; 12.5, 25, 50 and 100 mg/L in drinking water for four weeks; 0%, 0%, 16.7% and 33.3% mortality, respectively; lack of mortality as a dose selection criterion), a sub-lethal concentration of 25 mg/L was chosen as it had no long-term effect on clinical condition. Free-living birds can experience much higher diquat concentrations for short periods, since concentration as high as 22.2 g/L are used in a spray form for a weed control. Nevertheless, our intention here was to imitate a mild but long-term increase in oxidative load, such that may result for example from stressful conditions or chronic infection^7^,^57^. Considering that zebra finches drink ca. 2–4 mL water per day^55^, the concentration used in our experiment equals a daily intake of approximately 3–6 μg/g of body weight. A lethal dose for zebra finches is unknown, but our dose is much lower than LD50 reported for mallards (564 μg/g)^58^. During the main experiment, thirteen diquat-treated birds showed mild apathy and ruffled feathers at the beginning of the treatment (Yates’ chi-square tests: ROS effect, *χ*^*2*^ = 14.14, *P* < 0.001; carotenoid effect, *χ*^*2*^ = 0, *p* = 1). Aside from two birds from the ROS + CAR− group that died 35- and 43-days after the beginning of the experiment, and two birds from the ROS + CAR + group that died after 2 and 41 days, most birds recovered after a few days. There was no significant difference in mortality in diquat- or carotenoid-treated birds compared to control ones (Yates’ chi-square tests: ROS, *χ*^*2*^ = 2.41, *p* = 0.12; CAR, *χ*^*2*^ = 0.27, *p* = 0.61).

For the increase in carotenoid intake (CAR + groups), 1 kg of hulled millet seed was coated with 1 mL of lutein and zeaxanthin (FloraGLO Lutein 20% SAF, Kemin/DSM, France) mixed with 1 mL of safflower oil (Jules Brochenin, France). We used the highest known dose assimilated by zebra finches (200 μg of carotenoids per g of food)^35^ in order to test for the hypothesised pro-oxidant effect of high carotenoid levels under elevated oxidative stress^16^. Given that FloraGLO contains 10 mg/mL α-tocopherol as an antioxidant, the control (CAR−) diet was prepared by mixing 1 kg of seed with 2 mL of safflower oil and 10 mg of α-tocopherol (T3251, Sigma-Aldrich, Czech Republic). All prepared diets were frozen at −80 °C until use.

Pre- and post-experimental values for body mass and beak reflectance were taken and blood samples (up to 120 μL) collected from the jugular vein at the beginning and end of the experiment in heparinised microhaematocrit capillaries. The blood samples were centrifuged at 9,000 × g for 5 min and the plasma and RBC separated and stored at −80 °C for biochemical analysis. There was no difference in size (tarsus length) and size-controlled body mass (tarsus length as covariate) between experimental groups prior to the experiment (*p* ≥ 0.12).

### Beak colour measurement

Beak reflectance was measured between 300–700 nm on an AvaSpec 2048 spectrophotometer with an AvaLight-XE light source (Avantes, Netherlands). Four points were measured on each side of the upper beak, and two on the lower beak, with the probe held perpendicular to the surface. The spectrophotometer was standardised against a darkroom and a WS-2 white standard after every five individuals. Subsequent reflectance data processing and colour analysis was undertaken using the ‘pavo’ package in R 3.0.2^59^. All 12 spectral curves from each individual were interpolated to 1-nm steps, merged into one average curve and smoothed with a span value of 0.15. The resulting average curves were used to calculate colorimetric measures (hue, red chroma and UV chroma) and avian visual modelling. Hue was calculated as wavelength at the reflectance midpoint, whereas red and UV chroma were calculated as a summed reflectance of 600–700 nm and 300–400 nm, respectively, divided by a summed reflectance of 300–700 nm (formulas H3, S1R and S1U in^60^, red chroma modified according to^54^). Repeatability of red, UV chroma and hue measurements (*n* = 25) were 0.83, 0.50 and 0.91, respectively. While red chroma was strongly correlated with both hue (Spearman’s *r*_*P*_ = 0.76) and UV chroma (*r*_*P*_ = −0.88), the correlation between hue and UV chroma was somewhat weaker (*r*_*P*_ = −0.45). There was no between-group difference in either colour metric prior to the experiment (*p* ≥ 0.35). As both hue and logit transformed (see statistical analysis) UV chroma did not meet the assumption of normality (Shapiro-Wilk test: W = 0.958, *p* = 0.039 and W = 0.862, *p* < 0.001, respectively), logit transformed red chroma was used as a representative variable throughout the study and only report the effect sizes for both hue and UV chroma in the Supplementary Table S3.

### Plasma carotenoids

Total carotenoid content in plasma was assessed as the absorbance of an organic extract prepared according to the slightly modified method of McGraw *et al*.^52^. Briefly, 100 μL of ethanol was added to 10 μL of the plasma and the mixture then extracted with 100 μL of hexane:*tert*-buthyl methyl ether (1:1, v/v) by vortexing. After centrifugation (2 min at 11,000 × g), the upper organic layer was transferred to a 1.5 mL vial and evaporated to dryness under a stream of nitrogen. The dry sample was dissolved in 200 μL acetonitrile and 150 μL of this solution was pipetted into a 96-well microplate. Absorbance was measured at 450 nm (local absorption maximum of the plasma extracts) on an Infinite M200 microplate-reader (Tecan, Austria). Total plasma carotenoids were expressed as an equivalent of lutein concentration, the plasma carotenoid most prevalent in the zebra finch^35^. We used this measure in our analysis in order to provide information on the amount of all the carotenoids present in the plasma. In order to control whether this measure indeed correlated with the most prevalent plasma carotenoids, we also analysed plasma lutein and zeaxanthin concentrations using HPLC (see Supplementary Information). Both, lutein and zeaxanthin, were highly correlated with our estimate of total plasma carotenoids (*r* = 0.84 and 0.79, respectively), as well as to each other (*r* = 0.90). There was no difference in either lutein, or zeaxanthin, or total plasma carotenoids between the groups prior to the experiment (*p* ≥ 0.86).

### Plasma antioxidant capacity

The OXY-Adsorbent test (OXY; Diacron, Italy) was used as a measure of hydrophilic antioxidant capacity in blood plasma^61^. Five microliters of plasma diluted 1:100 with double-distilled water were incubated with 200 μL of HClO-based solution for 10 min at 37 °C. After incubation, the chromogen (5 μL of *N*,*N*-diethyl-*p*-phenylenediamine solution) was added and absorbance read immediately using a Sunrise microplate-reader (Tecan, Austria) at 505 nm. The antioxidant capacity of the sample was expressed as μmol of HClO neutralised by antioxidants present in 1 mL of the sample^61^. Repeatability of the OXY measurement was 0.76. There was no difference in OXY between the groups prior to the experiment (*p* = 0.12).

### Oxidative damage and lipophilic antioxidant capacity in RBC

The total RBC concentrations of 8-iso-prostaglandin F_2α_ (8-isoprostane), a product of arachidonic acid oxidation, was used as markers of total oxidative damage^22^. In addition, the ratio of HODE stereoisomeric forms (*ZE*/*EE*-HODE) was used as a measure of *in vivo* antioxidant capacity in lipophilic cellular compartments^21^,^22^. We expressed the relative amount of *ZE*- and *EE*-HODE stereoisomers as a proportion of *ZE*-HODE to total HODE (*ZE*/tHODE), rather than the *ZE*/*EE*-HODE ratio^21^, as proportional variables can easily be normalised through logit transformation and analysed with linear models. If necessary, the *ZE*/tHODE proportion can easily be converted to a *ZE*/*EE*-HODE ratio.

Details of the HPLC-ESI-MS/MS methods for 8-isoprostane and HODE analysis are described in Supplementary Information. The experimental groups did not differ in oxidative damage prior to the experiment (*p* ≥ 0.37). Despite random assignment of the birds to the experimental groups, however, we detected a significant difference in *ZE*/tHODE with the ROS−CAR + group having lower values (mean difference ± SE = −0.074 ± 0.029, *p* = 0.012) and the ROS + CAR + group higher (0.083 ± 0.029, *p* = 0.005) relative to the control group prior to the experiment. Although these differences may have influenced the effect size of the treatments, we believe that they do not change the general interpretation of our results as carotenoid supplementation produced a qualitatively similar effect in both groups, despite initial differences. Moreover, we used ANCOVA models that control for potential differences in initial values of the response variable.

### Statistical analysis

The effect of experimental manipulation was tested using general linear models implemented in R 3.1.2 (The R Foundation for Statistical Computing, Austria). In each model, diquat and carotenoid treatment were included as factors, together with their interaction, and pre-experiment (initial) values of the modelled trait were included as a covariate. Factor levels were coded as 0 and 1 (low and high, respectively) and centred to enable interpretation of the main effects without the need to remove the interaction^62^. Normalising logit (R package ‘car’) and Box-Cox (R package ‘MASS’) transformations were used for proportional variables (red and UV chroma and *ZE*/tHODE) and plasma carotenoids (total carotenoids and lutein), respectively. All models in our study were also run with continuous variables *z*-transformed in order to obtain standardised partial regression coefficients (b^*^) as a measure of standardised effect size^62^.

## Additional Information

**How to cite this article**: Tomášek, O. *et al*. Opposing effects of oxidative challenge and carotenoids on antioxidant status and condition-dependent sexual signalling. *Sci. Rep.* **6**, 23546; doi: 10.1038/srep23546 (2016).

## Supplementary Material

Supplementary Information

## Figures and Tables

**Table 1:** Effects of experimental manipulations on beak colouration, plasma carotenoids, body mass and blood redox state.

Response variables Predictors	Parameter estimates		Model statistics		Standardised effect size		
	Mean effect	SE	*t*	*P*	b^*^	CI 2.5%	CI 97.5%
Beak red chroma							
initial	0.17	0.10	1.64	0.11	0.13	−0.03	0.28
ROS	−0.26	0.05	−4.81	<0.001	−0.73	−1.04	−0.43
CAR	0.50	0.05	9.38	<0.001	1.44	1.13	1.75
ROS × CAR	0.07	0.11	0.61	0.54	0.19	−0.43	0.80
Total plasma carotenoids							
initial	0.08	0.02	4.35	<0.001	0.31	0.17	0.45
ROS	−0.71	0.24	−2.99	0.005	−0.42	−0.70	−0.14
CAR	2.64	0.24	11.08	<0.001	1.56	1.28	1.84
ROS × CAR	0.29	0.48	0.62	0.54	0.17	−0.39	0.74
*ZE*/tHODE ratio							
initial	−0.36	0.25	−1.44	0.16	−0.06	−0.15	0.02
ROS	0.85	0.04	20.10	<0.001	1.55	1.39	1.71
CAR	−0.57	0.04	−14.88	<0.001	−1.04	−1.18	−0.90
ROS × CAR	−0.42	0.09	−4.86	<0.001	−0.77	−1.08	−0.45
OXY							
initial	0.67	0.13	5.19	<0.001	0.60	0.37	0.83
ROS	20.56	7.29	2.82	0.007	0.64	0.19	1.11
CAR	−1.15	7.05	−0.16	0.87	−0.04	−0.48	0.41
ROS × CAR	4.13	14.17	0.29	0.77	0.13	−0.77	1.03
8-isoprostane in RBC							
initial	0.41	0.09	4.48	<0.001	0.53	0.29	0.76
ROS	1.97	1.04	1.89	0.07	0.43	−0.03	0.90
CAR	1.56	1.06	1.47	0.15	0.34	−0.13	0.81
ROS × CAR	−1.35	2.12	−0.64	0.53	−0.30	−1.24	0.64
Body mass							
initial	1.01	0.11	9.62	<0.001	0.83	0.66	1.00
ROS	−0.02	0.31	−0.06	0.95	−0.01	−0.33	0.31
CAR	−0.26	0.33	−0.80	0.43	−0.14	−0.48	0.21
ROS × CAR	−1.44	0.62	−0.62	0.02	−0.75	−1.40	−0.11

Estimates represent coefficients from linear models with oxidative challenge (ROS) and carotenoid intake (CAR) included as factors and pre-treatment (initial) values as covariates. Low and high factor levels were coded 0 and 1, respectively and centred in order to enable the main effects to be properly interpreted without the need to remove the interaction terms from the models. Proportional variables (i.e. beak red chroma and *ZE*/tHODE ratio) and total plasma carotenoids were normalised using logit and Box-Cox (λ = 0.335) transformation, respectively. Standardised effect sizes are reported as standardised partial regression coefficients (b^*^) from the same models with continuous variables *z*-standardised.
