# Supplementary material for: Opposing effects of oxidative challenge and carotenoids on antioxidant status and condition-dependent sexual signalling
Source: Sci Rep. 2016 Mar 22;6:23546. doi: 10.1038/srep23546 (PMC4802301; doi:10.1038/srep23546)
Supplement: Supplementary Information [file srep23546-s1.pdf]

Supplementary Information

for

Opposing effects of oxidative challenge and carotenoids on antioxidant status and condition-dependent sexual signalling

Oldřich Tomášek, Barbora Gabrielová, Petr Kačer, Petr Maršík, Jana Svobodová, Kamila Syslová, Michal Vinkler, Tomáš Albrecht

Supplementary inventory

Supplementary Data

Supplementary Table S1. Group mean values and SD of measured variables at the beginning of the experiment.

Supplementary Table S2. Group mean values and SD of measured variables at the end of the experiment.

Supplementary Table S3. Standardised effect sizes of oxidative load (ROS) and carotenoid intake (CAR) on beak hue and UV chroma and plasma lutein and zeaxanthin.

Supplementary Methods

Supplementary Table S4. Validation parameters of the LC-MS methods for determination of HODEs and 8-iso-prostaglandin $F_{2\alpha}$

Supplementary Table S1. Group mean values and SD of measured variables at the beginning of the experiment.

**Treatment group**	**ROS− CAR−**		**ROS− CAR+**		**ROS+ CAR−**		**ROS+ CAR+**	
**Variable**	**N**	**Mean (SD)**	**N**	**Mean (SD)**	**N**	**Mean (SD)**	**N**	**Mean (SD)**
Beak red chroma	15	0.85 (0.04)	15	0.85 (0.03)	15	0.85 (0.03)	15	0.86 (0.03)
Beak hue (nm)	15	590.33 (2.87)	15	590.20 (2.21)	15	588.93 (3.76)	15	589.07 (3.37)
Beak UV chroma	15	0.018 (0.022)	15	0.015 (0.018)	15	0.011 (0.012)	15	0.007 (0.009)
Total plasma carotenoids (µg/mL)	14	27.43 (3.18)	12	27.39 (8.36)	13	26.39 (5.18)	12	26.59 (7.93)
Plasma lutein (µg/mL)	14	18.06 (3.17)	12	17.70 (4.52)	13	17.08 (3.01)	12	17.73 (5.40)
Plasma zeaxanthin (µg/mL)	14	1.32 (0.85)	10	1.57 (1.07)	13	1.21 (0.56)	10	1.36 (1.43)
8-isoprostanes in RBC (pg/mL)	15	33.49 (6.93)	14	37.06 (6.57)	15	34.90 (3.71)	14	36.20 (5.09)
ZE/tHODE ratio	15	0.44 (0.02)	14	0.42 (0.02)	15	0.44 (0.02)	14	0.46 (0.01)
OXY (µmol HClO/mL)	15	138.76 (30.16)	15	139.00 (26.52)	15	118.93 (27.28)	15	124.23 (27.02)
Body mass (g)	15	16.39 (1.65)	15	17.20 (1.62)	15	16.02 (1.40)	15	17.33 (1.34)

Supplementary Table S2. Group mean values and SD of measured variables at the end of the experiment.

**Treatment group**	**ROS− CAR−**		**ROS− CAR+**		**ROS+ CAR−**		**ROS+ CAR+**	
**Variable**	**N**	**Mean (SD)**	**N**	**Mean (SD)**	**N**	**Mean (SD)**	**N**	**Mean (SD)**
Beak red chroma	15	0.85 (0.02)	15	0.90 (0.01)	13	0.81 (0.03)	13	0.88 (0.03)
Beak hue (nm)	15	588.33 (2.50)	15	595.73 (2.09)	13	583.69 (3.59)	13	592.38 (4.54)
Beak UV chroma	15	0.010 (0.006)	15	0.003 (0.003)	13	0.020 (0.010)	13	0.005 (0.006)
Total plasma carotenoids (µg/mL)	15	27.44 (4.91)	14	55.76 (13.40)	13	19.91 (6.99)	12	48.84 (17.11)
Plasma lutein (µg/mL)	14	17.41 (3.46)	14	40.54 (9.63)	13	16.43 (5.17)	12	45.47 (15.02)
Plasma zeaxanthin (µg/mL)	14	1.66 (0.89)	14	2.51 (1.59)	10	1.27 (0.92)	11	2.88 (1.94)
8-isoprostanes in RBC (pg/mL)	15	27.41 (4.40)	15	30.54 (4.16)	13	30.46 (4.16)	10	31.65 (4.96)
ZE/tHODE ratio	15	0.42 (0.02)	15	0.34 (0.03)	13	0.67 (0.04)	10	0.47 (0.02)
OXY (µmol HClO/mL)	15	136.52 (29.97)	15	133.66 (28.21)	13	143.45 (42.42)	12	149.62 (25.32)
Body mass (g)	15	16.71 (1.85)	15	17.93 (1.75)	13	16.85 (1.55)	13	17.15 (2.41)

Supplementary Table S3. Standardised effect sizes of oxidative load (ROS) and carotenoid intake (CAR) on beak hue and UV chroma and plasma lutein and zeaxanthin. Effect sizes are given as standardised partial regression coefficients (*b**) from linear models with oxidative load and carotenoid intake as factors, initial values as covariates, and with continuous variables *z*-standardised. Low and high factor levels were coded 0 and 1, respectively and centred in order to enable the main effects to be properly interpreted without the need to remove the interaction terms from the models. Beak UV chroma and plasma lutein were normalised using logit and Box-Cox ($\lambda = 0.116$) transformation, respectively.

**Response variables**	**Standardised effect size**		
**Predictors**	b^*	**2.5% CI**	**97.5% CI**
**Beak hue**			
initial	0.25	0.10	0.40
ROS	−0.61	−0.91	−0.31
CAR	1.42	1.12	1.70
ROS × CAR	0.13	−0.45	0.72
**Beak UV chroma**			
initial	0.16	−0.06	0.37
ROS	0.66	0.23	1.08
CAR	−1.09	−1.51	−0.67
ROS × CAR	−0.20	−1.03	0.64
**Plasma lutein**			
initial	0.28	0.15	0.41
ROS	0.05	−0.21	0.31
CAR	1.67	1.41	1.93
ROS × CAR	0.22	−0.30	0.74
**Plasma zeaxanthin**			
initial	0.53	0.31	0.76
ROS	−0.16	−0.61	0.28
CAR	0.91	0.47	1.35
ROS × CAR	0.05	−0.83	0.94

Supplementary methods

HPLC analysis of carotenoids in plasma samples. Dried organic extract of the plasma (see Material and Methods) was dissolved in a mobile phase (methanol:acetonitrile 1:1, v/v) and injected (40 µl) into the HPLC. The HPLC system (Q-grad quaternary pump, Watrex, Czech Republic; Midas autosampler, Spark, The Netherlands) was equipped with a 4.6 x 250 mm 5 µm C30 Develosil RP-Aqueous separation column with a C30 Develosil precolumn (Nomura Chemical Co., Japan). Methanol (A) and acetonitrile (B) were used as mobile phases for gradient elution (starting with 20% B, ramped to 45% at 30 min, 100% B from 33 to 43 min, followed by 10 min of equilibration with 20% B). The flow was set to 1 mL/min and the column temperature set at 35 °C (Mistral column thermostat, Spark, The Netherlands). Absorption spectra (range 300–600 nm) were measured using a DAD UV6000LP spectrometer (Thermo Finnigan, USA). Carotenoids (adonirubin, astaxanthin, cryptoxanthin, lutein, zeaxanthin) were identified by comparison of retention times and spectra with commercially available standards (Sigma-Aldrich, Czech Republic; CaroteNature, Switzerland). Quantitation of known carotenoids was performed at 450 and 470 nm using external standards.

HPLC analysis of oxidative damage and lipophilic antioxidant capacity in RBC.

Standards (13-(*Z*,*E*)-HODE), 9-(*Z*,*E*)-HODE, 8-iso-prostaglandin $F_{2\alpha}$ (8-isoprostane), 9-HODE-d_4 and 13-HODE-d_4 and 8-iso-prostaglandin $F_{2\alpha}$-d_4) were obtained from Cayman Chemical Company (MI, USA). 9-(*E*,*E*)-HODE and 13-(*E*,*E*)-HODE were obtained from Larodan Fine Chemicals AB (Malmo, Sweden).

The pre-treatment step was performed according to the following procedure: internal standards 9-HODE-d_4, 13-HODE-d_4 and 8-iso-prostaglandin $F_{2\alpha}$-d_4 (each internal standard was 10 ng/50 µL of RBC), butylated hydroxytoluene (100 µM – antioxidant) and an extraction solution of methanol and acetonitrile (100 µL of methanol and 100 µL of acetonitrile/50 µL of RBC) were added to the RBC. The mixture and RBC were sonicated for 10 min at 4 °C. The sample was then centrifuged at 9,000 × g at a temperature of 4 °C and the supernatant subsequently separated. The supernatant was

dried by stripping with nitrogen then dissolved in methanol and water (25 µL, methanol:water – 70:30 v/v) and immediately analysed by HPLC-ESI-MS/MS.

The LC-ESI-MS/MS system consisted of an Accela 1250 LC chromatogram (Thermo Scientific, USA), an Open Accela autosampler (Thermo Scientific, USA) and a TSQ Vantage mass spectrometer (Thermo Scientific, USA). The analytes were separated on a 100 × 2.1 mm 1.7 µm C18 Kinetex column (Phenomenex, USA) with a mobile phase (solvent A: 5 mM ammonium acetate, Solvent B: acetonitrile, solvent C: methanol) in a gradient elution with a flow rate of 200 µL/min. The HPLC elution program was as follows: 75% A, 20% B, 5% C (3 min) → 50% A, 45% B, 5% C (linear increase over 40 min, held for 5 min) → 75% A, 20% B, 5% C (linear decrease over 1 min, held for 5 min). The column temperature was maintained at 30 °C and the sample temperature at 4 °C. Injection volume was 10 µL. The analytes were eluted with the following retention times: 9-(*ZE*)-HODE = 36.6 min, 9-(*EE*)-HODE = 39.2 min, 13-(*ZE*)-HODE = 35.4 min, 13-(*EE*)-HODE = 37.2 min, and 8-iso-prostaglandin $F_{2\alpha}$ = 9.4 min. A mass spectrometer equipped with electrospray ionisation (ESI) was used for detecting HODEs, 8-iso-prostaglandin $F_{2\alpha}$ and their deuterium labelled internal standards (9-HODE-d_4, 13-HODE-d_4, 8-iso-prostaglandin $F_{2\alpha}$-d_4) in negative ionisation mode (ESI^-). The selective reaction monitoring mode was used. The scan monitoring reactions (precursor ion → fragment ion) used for the analyses and their collision induced dissociated (CID) energy were as follows: m/z = 295.4 Da (corresponding with $[M-H]^-$ ions) → m/z = 171.5 Da (corresponding with fragmentation ion) (CID = 22eV) for 9-(*ZE*)-HODE and 9-(*EE*)-HODE; m/z = 299.4 Da → m/z = 175.5 Da (CID = 22eV) for 9-HODE-d_4; m/z = 295.4 Da → m/z = 194.6 Da (CID = 21eV) for 13-(*ZE*)-HODE; and 13-(*EE*)-HODE , m/z = 299.4 Da → m/z = 198.6 Da (CID = 21eV) for 13-HODE-d_4; m/z = 299.4 Da → m/z = 198.6 Da (CID = 21eV); m/z = 353.5 Da → m/z = 193.1 Da (CID = 22eV) for 8-iso-prostaglandin $F_{2\alpha}$, and m/z = 357.5 Da → m/z = 197.1 Da (CID = 22eV) for 8-iso-prostaglandin $F_{2\alpha}$-d_4. The optimised conditions on the mass spectrometer were as follows: spray voltage = 2500 V, temperature of ion transfer tube = 300 °C, temperature of H-ESI vaporiser = 300 °C, pressure of sheath gas (nitrogen) = 35 psi, and flow of

auxiliary gas (nitrogen) set at 10 arbitrary units. The data were acquired and processed using Xcalibur v. 2.1.0 software (Thermo Scientific, USA).

The validation parameters (accuracy, precision, recovery and limit of detection and quantification) were assessed by analysing five different concentration levels (LOQ, 5, 10, 50 and 100 pg/μl) of the particular substances (9-(*ZE*)-HODE, 9-(*EE*)-HODE, 13-(*ZE*)-HODE, 13-(*EE*)-HODE, and 8-iso-prostaglandin $F_{2\alpha}$. The values of the validation parameters for each particular substrate were summarised in Supplementary table S2.

Table S4. Validation parameters of the LC-MS methods for determination of HODEs and 8-iso-prostaglandin F_{2a}

Analyte	Precision	Accuracy	Recovery	LOD	LOQ
	RSD (%)	RE (%)	(%)	(pg/μl)	(pg/μl)
9-(*ZE*)-HODE	16.7	−15.9	84.1	0.04	0.05
9-(*EE*)-HODE	16.5	−16.2	83.8	0.05	0.06
13-(*ZE*)-HODE	18.3	−14.8	85.2	0.06	0.07
13-(*EE*)-HODE	18.1	−15.0	85.0	0.05	0.06
8-iso-$PGF_{2\alpha}$	14.6	−12.8	87.2	0.08	0.09
